# Ischemic stroke management in West Scotland: a chart review

**DOI:** 10.3402/jmahp.v3.26339

**Published:** 2015-09-24

**Authors:** Patrice Verpillat, Julie Dorey, Chantal Guilhaume-Goulant, Firas Dabbous, Samuel Aballéa

**Affiliations:** 1 H. Lundbeck A/S, Paris, France; 2 Creativ-Ceutical, Paris, France; 3 Laboratoire Santé - Individu - Société (SIS, E.A. 4129), University of Lyon 1, Lyon, France; 4 Division of Epidemiology and Biostatistics, University of Illinois at Chicago, Chicago, IL, USA

**Keywords:** ischemic stroke, health-care resource utilization, thrombolysis, long-term management

## Abstract

**Background:**

Little information is available about the long-term management of ischemic stroke (IS) in West Scotland. In this study we aim to describe the management of IS at onset, admission, and during follow-up among patients who survived an IS event.

**Methods:**

General practitioners (GPs) (*n=*20) were randomly selected to recruit IS patients and extract data about patient characteristics, hospitalizations, discharge, and ambulatory care from GP databases, hospital letters, and direct contact with patients and their relatives. Descriptive analyses were conducted.

**Results:**

One hundred and one patients were included, with a mean age of 65.6±13.4. About half of the patients contacted their GPs at the time of onset (45.4%). Cardiovascular history was prevalent in 29.7% of cases, and 14% of all cases were recurrences. Of the patients, 89 (88%) were hospitalized with mean length of stay (LOS) 11.8 days. Treatment was administered on average within 12.9 hours of admission and 23.6% of the admitted patients received thrombolytic treatment. During the 1-year follow-up period, 33.6% of patients were rehospitalized and the mean LOS was 15.1±29.5 days. Further, patients on average sought nursing care (10.9%), physical therapy (45.5%), occupational therapy (27.7%), speech therapy (12.9%), and professional caregivers (12%).

**Conclusion:**

The health-care resource utilization of IS patients is a major driver of economic burden.

Ischemic stroke (IS) is the most common type of stroke among the Scottish population. It is a heterogeneous disease with 50% of cases attributed to high blood pressure. Older age, diabetes, and atrial fibrillation are also known risk factors (1). IS is the third cause of death in the United Kingdom; it claims the lives of 60,000 patients annually (2) and is a major cause of severe disability (3). It was estimated that around 110,000 first and recurrent stroke events occur in England every year (4). In Scotland, however, the risk of suffering and dying from stroke is higher compared to that in the rest of the United Kingdom. The Stroke Association estimated stroke incidence to be 202 for men and 160 for women per 100,000 in Scotland, compared to 178 and 139 in England, respectively. Prevalence was estimated to be 3.3% for men and 2.5% for women in Scotland, compared to 2.4 and 2.2% in the United Kingdom (5). In addition, stroke-specific cause of death is 41% higher in Scotland compared to the rest of the United Kingdom (6). The annual cost of stroke care was estimated to be £9 billion, with direct care accounting for 49%, informal care (defined as the time spent by family/friends or professional carers) for 27%, and indirect costs for 24%. Furthermore, around 200,000 IS individuals required assistance either from professional caregivers or from family members to carry out daily living activities, leading to a substantial economic burden on society (7).

In the United Kingdom, there are multiple organizations that provide guidance on stroke management to prevent strokes and reduce their burden, for example the Department of Health, which developed a national stroke strategy; the National Institute for Health and Clinical Excellence (NICE); and the evaluation unit of the Royal College of Physicians (8). The National Stroke Strategy focuses on raising awareness among both medical professionals and the general public but does not act as a detailed clinical guideline; rather it provides a quality framework for stroke services (9). Along with the National Stroke Strategy, the NICE guidelines focus on the acute phase; they recommend admission to an acute stroke unit directly from accident and emergency units and performing brain imaging as soon as possible. Treatment of acute IS using alteplase was approved by NICE but the guidelines did not specify the time window for administering the medication (10).

A few studies have described the management of IS patients, but most focus on the prehospitalization and acute phases. These studies describe arrival methods, time to arrival, length of stay (LOS), and transfer to units within the hospital. In the United Kingdom, the rate of arrival via ambulance was estimated at 50% (11), and the median time between onset of symptoms and hospital arrival was within 3 hours for 37% of patients and within 6 hours for 50% of the patients (12). Hospital admission rates ranged from 56% in Oxfordshire (13) and 83% in South London to 91% (14) in the Scottish Borders (15). Among those admitted to hospital, 25.4% were either admitted or transferred to the stroke unit (16). Although the mean LOS decreased from 23.7 days in 2008 to 19.5 in 2010, it is still longer compared to other European countries. Data from the National Sentinel Stroke Audit 2008 showed that the mean LOS on acute stroke units was 13 days (median 7 days), which was notably long.

## Objectives

The long-term management of IS stroke in West Scotland (WS) is not well documented in the literature; therefore we conducted this descriptive study in order to describe patient management and resource utilization during the first year following the occurrence of IS.

## Methods

A representative sample of 20 general practitioners (GPs) from WS was included in this retrospective chart review. The GPs identified and included, in a systematic way, IS patients from their databases. Patients with less than 1 year of follow-up and those who suffered from transient ischemic attack, hemorrhagic stroke, or stroke with undetermined reasons were excluded. IS events that occurred between 2007 and 2008 were included and data extraction took place between November 2009 and January 2010.

The GPs extracted information from the patients’ medical records with regards to 1) patient characteristics (age, sex, occupation, living condition, and medical history including stroke history, cardiovascular history, and previous ischemic event), 2) prehospitalization period (first health-care contacts, mode of arrival), 3) hospitalization (type of hospital, first unit admitted to, units transferred to within hospital, imaging procedures, time between admission and treatment, type of treatment, LOS in hospital in the different units, and severity scales), and 4) 1-year follow-up postdischarge (initial postdischarge period, rehospitalization, outpatient care sick leave, and change in occupation). Information regarding arrival method, type of hospital, first unit admitted to, units transferred to within hospital, imaging procedures (CT scans, MRI, ultrasound, or cerebral angiogram), time between admission and treatment, type of treatment, and LOS were documented in the letter that was sent to the GPs from each treating hospital. In addition, patients’ care providers were contacted to complete missing information.

## Results

A random sample of 20 GPs from a clinical research company network in West Scotland identified and recruited 101 IS cases that occurred between 2007 and 2008. The sampled patients were mostly living in an urban setting (92.1%), retired (74%), and female (52.5%) and were on average 65.6±13.4 years of age. Cardiovascular history was prevalent in 29.7% of the patients and 14% of the strokes were recurrences (Table 1). At the time of onset, almost all patients contacted a health-care provider (96%); however, 45.4% of patients called their GP instead of the emergency hotline. Eighty-two patients were transported to a health-care facility via emergency vehicle (54.5%), their own car (21.7%), or other forms of transportation including taxis (3%) (Table 2).

**Table 1:** Patient characteristics

Characteristic	*N=*101
Mean age (SD)^a^	65.5 (13.4)
Males	48 (47.5)
Urban residence	93 (92.1)
Marital status	
Married/living with a partner	68 (67.3)
Single	12 (11.9)
Divorced/separated	5 (5)
Unknown	16 (15.8)
Current housing accommodation	
Home	80 (79.2)
Living with family	19 (18.8)
Shelter	2 (2)
Employed at the time of event	26 (25.7)
Medical condition	
Stroke history	14 (13.9)
At least one cardiovascular event	30 (29.7)
Ischemic event	3 (3)
Myocardial infarction	5 (5)
Angina pectoris	4 (4)
Carotid stenosis	1 (1)
Atrial fibrillation	3 (3)
Other cardiovascular history	14 (14)

a SD=standard deviation.

**Table 2:** Prehospitalization phase

	Total, *N=*101; *n* (%)
Type of health professional contacted at the time of event	
General practitioner (referring physician)	46 (45.4)
NHS 24	9 (8.9)
Accident & emergency department	11 (10.8)
Other	30 (29.7)
Other/NHS 24	1 (1.0)
Unknown	4 (4.0)
Mode of arrival to hospital	
Ambulance	55 (54.5)
Individual car	22 (21.7)
Taxi	3 (3)
Other	2 (2)
Unknown	19 (19)

## Hospitalization

Of the 101 patients, 89 (88%) were admitted to hospitals: specialized or university hospital (32.6%), general hospital (62%), or clinic or local hospital (2%). The first unit of admission was the emergency unit for 59.6% of patients and the stroke unit for 19% of patients. The average LOS in hospital was 11.8 days. During their hospitalization, 46% of patients were transferred to the stroke unit. Compliant with NICE guidelines, 84% of the admitted patients received brain imaging, such that 50% had a CT scan with or without contrast injection, 24% had an MRI, and 32% had a cervical Doppler ultrasound examination. Treatment was administered on average within 12.9 hours from admission and 23.6% of the patients received thrombolytic treatment. Other therapies such as re-adaptation care, physical therapy, and reeducation were also common during hospitalization (Table 3).

**Table 3:** Hospitalization and health-care resource utilization at acute phase

	Total, *N* a=89; *n* (%)
Hospitalization	
Length of stay in days (LOS) in hospital, mean (STD)	11.8 (17.7)
Time in hours between admission and treatment, mean (STD)	12.9 (5.8)
Type of hospital	
Specialized hospital	29 (32.6)
General hospital	55 (61.8)
Clinic/local hospital	2 (2.2)
First hospital unit of admission	
Emergency unit	53 (59.6)
Stroke unit	17 (19.1)
Intensive care unit	1 (1.1)
Neurology unit	1 (1.1)
General medicine	5 (5.6)
Geriatrics	2 (2.2)
Cardiology	1 (1.1)
Other units	9 (10.1)
Imaging test	
Number of imaging tests	75 (84.3)
Type of imaging testing	
CT scan without contrast injection	28 (37.3)
CT scan with contrast injection	10 (13.3)
MRI	18 (24)
Cerebral angiography, angioscan, or Magnetic Resonance Angiogram (MRA)	1 (1.3)
Cervical Doppler ultrasound examination	24 (32)
Thrombolysis treatment	21 (23.6)

a 89 out of the 101 patients were hospitalized.

## Follow-up phase

At discharge, patients’ options varied between going to their homes (85.4%), using home medical care (12%), going to rehabilitation centers (4.5%), and staying with family or friends (4.5%). During the 1-year follow-up period, 33.6% of patients were rehospitalized and the mean LOS was 15.1±29.5 days. On average, patients made 5.5 visits to their GP, 6.46 visits to a psychologist or psychiatrist, and 1.2 visits to a neurologist. Further, patients sought nursing care (10.9%), physical therapy (45.5%), occupational therapy (27.7%), speech therapy (12.9%), and professional caregivers (12%). Of the employed patients, 76.9% reported taking sick leave (Table 4). Mortality data was not available.

**Table 4:** Health-care resource utilization during the chronic phase (1-year follow-up)

	*N=*101; *n* (%)
Living situation immediately after hospital discharge^a^	
Home^b^	76 (85.4)
Rehabilitation center^b^	4 (4.5)
Home medical care^b^	11 (12)
Staying with relative or friend^b^	4 (4.5)
Mean length of stay in days	
Home	6.7 (1.1)
Rehabilitation center	35 (27.4)
Staying with relative or friend	34.8 (57.1)
Home medical care	27.8 (51.6)
Rehospitalization	34 (33.6)
Rehospitalization – LOS in days	15.1 (29.5)
Health care utilization	
Outpatient visits^a^	
Psychologist and psychologist support visits^a^	18 (17.8)
Psychiatrist visits^a^	4 (3.9)
Psychotherapist visits^a^	4 (3.9)
Nursing care^a^	11 (10.9)
Physiotherapist visits^a^	46 (45.5)
Occupational therapy visits^a^	28 (27.7)
Speech therapy visits^a^	13 (12.9)
Mean number of neurologist visits^c^	1.2 (1.5)
Mean number of general practitioner visits^c^	5.5 (5.6)
Mean number of psychologist support visits^c^	6.5 (4.9)
Mean number of nursing care visits per week^c^	3.3 (2.7)
Mean number of physiotherapist visits per week^c^	2 (1.4)
Mean number of occupational therapy visits per week^c^	1.9 (1.3)
Mean number of speech therapy visits per week^c^	1.5 (0.9)
Sick leave	20 (76.9)
Changed occupation	5 (19.2)
Change to part time job	6 (23.0)
Caregiver characteristics	
Caregiver	36 (35.6)
Professional	12 (33.3)
Personal	30 (29.7)
Still receiving follow-up care by general practitioner	19 (18.8)

a Including first place post event for non-hospitalized patients;

b proportion of hospitalized patients;

c among patients with at least one visit.

## Discussion

To our knowledge, this is the first study to follow such a design and recruit IS patients through GPs in WS. However, this approach, known as chart reviews, has been used in other disease areas. For instance, a study with a similar design was conducted in Germany in which the authors extracted information about heart failure patients’ medication from the hospital discharge letter and from the GPs via phone interviews; the authors also surveyed the patients to capture their actual medication use during the 14-day follow-up (17).

In addition, this study is the first to describe the characteristics of the patients and management during the acute phase and to assess and describe the management and health-care resource utilization postdischarge during a 12-month follow-up period. GPs usually manage IS patients over the long term after hospital discharge and are well informed via hospital letter about their patients’ hospital stay, all procedures performed, and treatment administered. They are also the referring physicians. These attributes allow GPs to identify and recruit patients as well as to collect and report information during the preadmission phase, hospital stay, and postdischarge of IS patients.

Although the sample of patients we recruited was relatively modest, in this study we were able to describe the options for patients after discharge and their disease management during all phases of their condition. Firstly, almost half of the patients (45.4%) contacted their GPs at the onset of symptoms instead of contacting the emergency hotline. This tendency may indicate a lack of knowledge about stroke symptoms among the general population, stressing the need to initiate campaigns to raise awareness of stroke in the WS population. Also, this study documents the fact that clinical severity scales such as the National Institutes of Health Stroke Scale might not be used during diagnosis of the IS patients and at discharge or at least such use is not reported to GPs. Consistent with the guidelines stating that all patients should receive imaging procedures using either CT or MRI within 24 hours of admission, 84% of this population received at least one form of imaging. Additionally, consistent with the National Sentinel Stroke Audit 2009 Organisational Audit Report, which reported an 89% hospitalization rate for IS patients, the vast majority of the patients in this study were admitted to the hospital (88%). The mean LOS among this sample was 11.8 days, which is comparable to that estimated in DRG (i.e., 10.7 days), but lower than published data (23.7 days in the United Kingdom [18]; 27 days in Scotland [19]). During the 1-year follow-up post initial stroke, 33% of the patients were hospitalized compared to 13.4% for the general population in Scotland (estimated for the 2009–2010 period using number of patients with hospital stays as the numerator and total Scottish population in 2010 as the denominator). This rate is slightly higher than that reported in the United States and the European Union, which reported readmission rate to be somewhere between 20 and 27% (20, 21). IS patients suffer from several health problems such as falls, depression, deterioration of physical and social activities, and other age-related health problems. In addition, about 13% (10–16% CI) of stroke patients may suffer from another episode of stroke within the first year, a rate that is 15 times higher than the general population (22). All these factors may explain the high rates of rehospitalization.

Finally, even though the thrombolysis rate may not be accurate, this study highlights the fact that many patients who are eligible to receive thrombolysis within the recommended time window do not receive it. The approach used in this study has several limitations that should be mentioned. First, the method used to select patients may not ensure the generalizability of the patients, as the GPs only included patients who were alive at the time of the study. Furthermore, some GPs did not have a database or electronic medical records for their patients and they recruited patients relying on their memory. Consequently, not all patient characteristics were consistent with the published literature. For instance, the mean age of this sample (65 years) was lower than that estimated by Langhorne et al. (mean age 75) and by Lewsey et al. (mean age for men was 71 and 76 among women). This age distribution may also explain the elevated rate of thrombolysis (23%) among our population compared to the national average (3 and 5% in Scotland for 2008 and 2009, respectively) (23), because younger patients are more eligible for recombinant tissue plasminogen activator and have a higher chance of benefitting from the treatment.

In addition, mortality data was not reported due to the method used to recruit patients. Secondly, the selection of GPs from the network could explain the high rate of patients from an urban setting (92%), as it is more embedded in urban settings. Furthermore, the hospital letter received by GPs following hospital discharge does not include detailed information with regards to severity scales used to assess the condition of the patient at the acute phase. Moreover, severity scales were not reported in the charts. During the interviews, the GPs informed us that severity indices are either used in the hospital but results are not released or they are used in stroke units only.

In conclusion, we described in general terms the postdischarge health-care resource utilization of IS patients, which is a major driver of economic burden in the long-term care of IS patients.

## References

[1] Lawes CM, Vander HS, Rodgers A (2008). Global burden of blood-pressure-related disease, 2001. Lancet.

[2] (2007). Stroke Prevention & Treatment: Turning Knowledge into Practice. A Report from the ‘Action Group on Stroke is a Catastrophic Disease’.

[3] Shannon C (2004). Stroke services have improved but remain overstretched. BMJ.

[4] National Institute for Health and Clinical Excellence (2015). Stroke: Diagnosis and initial management of acute stroke and transient ischaemic attack (TIA).

[5] Stroke Association (2013). Stroke statistics 2013.

[6] McCartney G, Russ TC, Walsh D, Lewsey J, Smith M, Smith GD (2015). Explaining the excess mortality in Scotland compared with England: pooling of 18 cohort studies. J Epidemiol Community Health.

[7] Saka O, McGuire A, Wolfe C (2009). Cost of stroke in the United Kingdom. Age Ageing.

[8] Royal College of Physicians (2012). National clinical guideline for stroke.

[9] Department of Health (2007). National stroke strategy.

[10] NICE (2008). Stroke: diagnosis and initial management of acute stroke and transient ischaemic attack (TIA).

[11] Deakin CD, Alasaad M, King P, Thompson F (2009). Is ambulance telephone triage using advanced medical priority dispatch protocols able to identify patients with acute stroke correctly?. Emerg Med J.

[12] Harraf F, Sharma AK, Brown MM, Lees KR, Vass RI, Kalra L (2002). A multicentre observational study of presentation and early assessment of acute stroke. BMJ.

[13] Rothwell PM, Coull AJ, Giles MF, Howard SC, Silver LE, Bull LM (2004). Change in stroke incidence, mortality, case-fatality, severity, and risk factors in Oxfordshire, UK from 1981 to 2004 (Oxford Vascular Study). Lancet.

[14] Stewart JA, Dundas R, Howard RS, Rudd AG, Wolfe CD (1999). Ethnic differences in incidence of stroke: prospective study with stroke register. BMJ.

[15] Syme PD, Byrne AW, Chen R, Devenny R, Forbes JF (2005). Community-based stroke incidence in a Scottish population: the Scottish Borders Stroke Study. Stroke.

[16] McKevitt C, Coshall C, Tilling K, Wolfe C (2005). Are there inequalities in the provision of stroke care? Analysis of an inner-city stroke register. Stroke.

[17] Scherer M, Sobek C, Wetzel D, Koschack J, Kochen MM (2006). Changes in heart failure medications in patients hospitalised and discharged. BMC Fam Pract.

[18] Langhorne P, Lewsey JD, Jhund PS, Gillies M, Chalmers JW, Redpath A (2010). Estimating the impact of stroke unit care in a whole population: an epidemiological study using routine data. J Neurol Neurosurg Psychiatry.

[19] Lewsey JD, Jhund PS, Gillies M, Chalmers JW, Redpath A, Kelso L (2009). Age- and sex-specific trends in fatal incidence and hospitalized incidence of stroke in Scotland, 1986 to 2005. Circ Cardiovasc Qual Outcomes.

[20] Thorngren M, Westling B, Norrving B (1990). Outcome after stroke in patients discharged to independent living. Stroke.

[21] Sacco RL, Hauser WA, Mohr JP (1991). Hospitalized stroke in blacks and Hispanics in northern Manhattan. Stroke.

[22] Burn J, Dennis M, Bamford J, Sandercock P, Wade D, Warlow C (1994). Long-term risk of recurrent stroke after a first-ever stroke. The Oxfordshire Community Stroke Project. Stroke.

[23] Clinical Effectiveness and Evaluation Unit Royal College of Physicians of London (2009). National Sentinel Stroke Audit Phase II (clinical audit) 2008.

